# MicroRNA-100-5p indirectly modulates the expression of *Il6*, *Ptgs1/2* and *Tlr4* mRNA in the mouse follicular dendritic cell-like cell line, FL-Y

**DOI:** 10.1111/imm.12342

**Published:** 2014-12-08

**Authors:** Susan R Aungier, Hitoshi Ohmori, Michael Clinton, Neil A Mabbott

**Affiliations:** 1 The Roslin Institute and R(D)SVS, University of EdinburghMidlothian, UK; 2 Department of Bioscience and Biotechnology, Okayama University Graduate School of Natural Science and TechnologyOkayama, Japan

**Keywords:** follicular dendritic cell, microRNA, mmu-miR-100-5p, mRNA, spleen

## Abstract

Follicular dendritic cells (FDC) are important stromal cells within the B-cell follicles and germinal centres (GC) of secondary lymphoid tissues. FDC trap and retain native antigens on their surfaces in the form of immune complexes that they display to B cells, in order to select those cells with the highest antigen affinity. MicroRNAs are short, non-coding RNAs that are approximately 18–25 nucleotides in length that regulate gene expression at the post-transcriptional level by repressing the translation of target genes. In the current study, *in vivo* and *in vitro* systems were used to identify microRNAs that were potentially expressed by FDC. Constitutive lymphotoxin-*β* receptor (LT*β*R) stimulation is required to maintain FDC in their differentiated state. We show that the rapid de-differentiation of spleen FDC that accompanied LT*β*R-blockade, coincided with a significant decrease in the expression of mmu-miR-100-5p, mmu-miR-138-5p and mmu-miR-2137. These microRNAs were shown to be expressed in the FDC-like cell line, FL-YB, and specific inhibition of mmu-miR-100-5p significantly enhanced expression of *Il6*, *Ptgs1/2* and *Tlr4* mRNA in this cell line. The expression of *Il6*, *Ptgs1/2* and *Tlr4* by FDC play important roles in regulating GC size and promoting high-affinity antibody responses, so it is plausible that mmu-miR-100-5p may help to regulate the expression of these genes during GC reactions.

## Introduction

Follicular dendritic cells (FDC) are a unique population of non-migratory, non-phagocytic stromal cells.^1^–^3^ These cells have long dendrites that are used to form large networks within the B-cell follicles and germinal centres (GC) of secondary lymphoid tissues such as the spleen and lymph nodes. FDC express complement receptors (CR; CR1/CD35 and CR2/CD21) and immunoglobulin Fc receptors (e.g. Fc*γ*RIIb), which they use to capture antigen in the form of immune complexes (antigen–antibody and/or opsonizing complement components) on their surfaces.^4^ Antigens are retained on FDC in a native state for long periods of time to promote immunoglobulin-isotype class switching, the positive selection of B cells with high antigen affinity and the maintenance of immunological memory.^5^,^6^ The up-regulation of adhesion molecules by FDC further facilitates interactions between B cells and the FDC-retained antigens.^7^ The competitive binding of antigen on the FDC surface in the GC allows those B cells with the highest specific antigen affinity to receive pro-survival signals from FDC via cytokines such as interleukin-6 (IL-6) and IL-15, whereas those with low antigen affinity are targeted for their clearance via apoptosis.^8^,^9^ In addition, FDC also secrete the chemokine CXCL13, which stimulates the chemotaxis of CXCR5-expressing B cells into follicles and GC.^10^,^11^ The secretion of milk fat globule epidermal growth factor-like factor 8 (MFGE8) by FDC mediates the engulfment of apoptotic B cells within the GC by tingible body macrophages.^8^

In the secondary lymphoid tissues FDC develop from precursors cells surrounding the blood vessels and they require further stimulation from lymphoid tissue inducer cells and lymphotoxin (LT) for expansion.^2^ FDC and their precursor cells express the LT*β* receptor (LT*β*R) and stimulation through this receptor by B-cell-derived LT*α*_1_*β*_2_ maintains FDC in their differentiated state. In the absence of constitutive LT*β*R-mediated stimulation FDC rapidly de-differentiate.^12^,^13^

MicroRNAs are short, non-coding RNA species, approximately 18–25 nucleotides in length, that act by targeting partially complementary sequences within mRNAs and most commonly lead to translational repression and/or degradation of the target mRNAs.^14^ There are over 1000 different microRNAs expressed in human tissues and each microRNA is thought to target hundreds of genes. Consequently, microRNAs are predicted to regulate the activity of approximately 50% of all known protein-coding genes in mammals. MicroRNAs are most commonly transcribed as independent genes or from protein-coding genes, and microRNA genes may be clustered within specific genomic regions.^14^ MicroRNA transcripts are processed into stem-loop microRNA precursors by the RNAse III endonuclease DROSHA, and then exported to the cytoplasm where a second endonuclease, Dicer, cleaves the pre-microRNA into the mature form. Mature microRNAs are assembled into ribonucleoprotein complexes where they interact with specific mRNA targets. MicroRNAs are often transcribed in a tissue-specific and developmental stage-specific manner,^15^ and a small number have been described as master regulators of developmental processes.

Abundant expression of microRNAs has been described in many immune cell populations and has been shown to be involved in the regulation of a number of immune functions.^16^–^18^ For example, studies in mice show that within the GC microRNAs play a role in the regulation of gene expression by B cells and follicular helper T cells.^19^ Although available data suggest that human FDC-like cells (HK cells) may induce the expression of certain microRNAs in GC B cells to promote survival,^20^ information on the expression of microRNAs by FDC has not previously been reported. Similarly, nothing was known of the effects of LT*β*R stimulation on microRNA expression in the spleen. Therefore, in the current study *in vivo* and *in vitro* systems were used to identify potential microRNAs that may modulate gene expression in FDC.

## Materials and methods

### 

#### Mice

Lymphotoxin-*β*-deficient (LT*β*^−/−^) mice^21^ were obtained from B & K Universal Ltd (Hull, UK) and were maintained on a C57BL/6 background. Age-matched (5–7 weeks old) C57BL/6 mice were used as wild-type (WT) controls. Mice were housed under specific pathogen-free conditions with a 12-hr light/dark cycle and food and water were provided *ad libitum*. All experiments were conducted under the provisions of the UK Animals Scientific Procedures Act 1986 and approved by the University of Edinburgh's Ethical Review Committee.

#### In vivo LTβR blockade

Where indicated, C57BL/6 mice were given a single intravenous injection of 100 μg of a fusion protein comprising the soluble LT*β*R domain linked to the Fc portion of human IgG1 (LT*β*R-Ig)^22^ to temporarily deplete their FDC.^12^,^13^

#### Immunohistochemistry

Spleens were removed and divided into two approximately equal portions. One portion was used to generate RNA for downstream analyses, while the other was processed for histological analyses. Serial frozen sections (8 μm in thickness) were cut on a cryostat and immunostained with the following antibodies: FDC were visualized by staining with rat anti-CD35/CR1 monoclonal antibody (mAb) (clone 8C12; BD Biosciences PharMingen, Oxford, UK) or rat anti-MFGE8 mAb (clone FDC-M1; BD Biosciences PharMingen). B cells were detected using mAb B220 to detect CD45R (Caltag, Towcester, UK) and T cells were detected using mAb 17A2 to detect CD3 (Cambridge Bioscience, Cambridge, UK). For light microscopy, following the addition of primary antibodies, biotin-conjugated species-specific secondary antibodies (Stratech, Soham, UK) were applied followed by alkaline phosphatase coupled to the avidin/biotin complex (Vector Laboratories, Peterborough, UK). Vector Red (Vector Laboratories) was used as a substrate and sections were counterstained with haematoxylin to distinguish cell nuclei. For fluorescent microscopy, following the addition of primary antibody, species-specific secondary antibodies coupled to Alexa Fluor 488 (green) or Alexa Fluor 594 (red) dyes (Invitrogen, Paisley, UK) were used. Sections were mounted in fluorescent mounting medium (DakoCytomation, Ely, UK) and examined using a Zeiss LSM5 confocal microscope (Zeiss, Welwyn Garden City, UK).

#### Quantitative real-time PCR analysis of mRNA expression

Total RNA was isolated from spleens and cells using RNA-Bee (AMS Biotechnology, Oxfordshire, UK) followed by treatment with DNase I (Ambion, Warrington, UK). First-strand cDNA synthesis was performed using 1 μg of total RNA and the First-Strand cDNA Synthesis kit (GE Healthcare Life Sciences, Little Chalfont, UK) as described by the manufacturer. PCR amplification reactions were performed using the Platinum-SYBR Green qPCR SuperMix-UDG kit (Life Technologies, Paisley, UK) and the Stratagene Mx3000P real-time quantitative PCR system (Life Technologies). All quantitative PCR primers used were designed using primer3 software^23^ and their details are provided in the Supporting information (Table S1). The cycle threshold values were determined using mxpro software (Stratagene) and normalized to the value of *Actb* or *Hprt*.

#### Microarray analyses of microRNA expression

RNA prepared from four mice per group was split into two groups of two, and each group of two spleens was pooled. These RNA pools were then analysed in parallel as described below using Affymetrix and Exiqon microRNA expression arrays.

For Affymetrix, 2 μg of each RNA sample, prepared as above, was hybridized to Affymetrix GeneChip miRNA 2.0 expression arrays (Affymetrix, Santa Clara, CA). First the quality of the total RNA was verified by an Agilent 2100 Bioanalyzer profile (Agilent Technologies, Inc., Santa Clara, CA). Microarray analysis was performed by Edinburgh Genomics (University of Edinburgh, UK) using Affymetrix systems and reagents. Hybridization probes were generated using the Ambion WT labelling kit and 500 ng of RNA. Signal intensities were measured using a GeneChip Scanner 3000 7G and CEL files were generated using GeneChip Command Console software (AGCC). Data were background corrected and normalized by robust multi-array analysis using the ExiMiR package in R/Bioconductor.^24^

For Exiqon, 5 μg of each RNA sample was sent to Exiqon (Denmark) for quality analysis and subsequent microRNA microarray analysis. The quality of the total RNA was verified by an Agilent 2100 Bioanalyzer profile. A 750-ng subsample of total RNA from both sample and reference was labelled with Hy3™ and Hy5™ fluorescent label, respectively, using the miRCURY LNA™ microRNA Hi-Power Labeling Kit, Hy3™/Hy5™ (Exiqon, Vedbaek, Denmark) following the procedure described by the manufacturer. The Hy3™-labelled samples and a Hy5™-labelled reference RNA sample were mixed pair-wise and hybridized to the miRCURY LNA™ microRNA Array 6th GEN (Exiqon), which contains capture probes targeting all microRNAs for human, mouse or rat registered in the miRBASE 16.0. The hybridization was performed according to the miRCURY LNA™ microRNA Array Instruction manual using a Tecan HS4800™ hybridization station (Tecan, Männedorf, Switzerland). The miRCURY LNA™ microRNA Array slides were scanned using the Agilent G2565BA Microarray Scanner System and the image analysis was carried out using the ImaGene® 9 (miRCURY LNA™ microRNA Array Analysis Software; Exiqon). The quantified signals were background corrected (Normexp with offset value 10)^25^ and normalized using the global Lowess (LOcally WEighted Scatterplot Smoothing) regression algorithm.

Differentially expressed microRNAs were determined using the limma package in R/Bioconductor.^26^ All raw microarray data (CEL files) are deposited in the Gene Expression Omnibus (GEO) under the following accession number: GSE55722. (http://www.ncbi.nlm.nih.gov/geo/query/acc.cgi?acc=GSE55722).

#### microRNA Northern blot analysis

The expression profiles of selected microRNAs identified by the microarray analyses (above) were validated by microRNA Northern blot analysis. RNA was separated by 15% polyacrylamide TBE–urea gel (Criterion; Bio-Rad, Hemel Hempstead, UK) electrophoresis at 125 V for 90 min and visualized using Sybr®Gold nucleic acid dye (Life Technologies). RNA was transferred to a nylon membrane (Amersham Hybond-NX; GE Healthcare Life Sciences) by semi-dry transfer at 220 mA for 1 hr. RNA was then cross-linked to the membrane using 1-ethyl-3-[3-dimethylaminopropyl]carbodiimide hydrochloride cross-linking solution according to the protocol described by Pall and Hamilton.^27^ For detection of specific microRNA molecules, membranes were pre-hybridized with ULTRAhyb Oligo buffer (Ambion) at 42° for a least 2 hr and then incubated overnight at 42° with complementary radiolabelled locked nucleic acid (LNA) -modified oligonucleotides in ULTRAhyb Oligo buffer (Ambion). LNA-modified oligonucleotides were obtained from Exiqon with LNA nucleotides placed every three nucleotides (see Supporting information; Table S2). Oligonucleotides were radiolabelled at the 5′ end with [*γ*-^32^P]ATP using a mirVana probe and marker kit (Ambion) according to manufacturer's protocol. After removal of unbound oligonucleotide, membranes were wrapped in saran wrap, placed in an exposure cassette and exposed to autoradiograph film at −80° in the presence of a maximum sensitivity intensifying screen for between 4 and 24 hr. Autoradiographs were developed using a Konica SRX-101A X-ograph machine. Membranes were routinely subjected to a series of washes and exposures to a final stringency of 0·1 × SSC, 0·1% SDS and 60°. To strip bound LNA-modified oligonucleotides, membranes were washed in boiling 0·1 × SSC and checked for residual signal by exposure to autoradiograph film. Membranes were then re-probed as appropriate for either other microRNAs of interest or for U6-snRNA as a loading control. To quantify hybridized radiolabelled nucleic acid after microRNA Northern blot analysis, membranes were exposed to phosphorimager screens at room temperature for the required length of time. For quantification screens were scanned using a Typhoon FLA 7000 system (GE Healthcare Life Sciences) and data were analysed using imagequant tl software (GE Healthcare Life Sciences).

#### Cell culture

The established FDC-like cell line, FL-YB^28^ was used. This cell line was originally derived from the popliteal lymph nodes of immunized BALB/c mice as described previously.^28^ FL-YB cells were cultivated in a 1 : 1 mixture of Dulbecco's modified Eagle's medium and RPMI-1640 media (Life Technologies) supplemented with 10% heat-inactivated fetal calf serum (Life Technologies), 1× penicillin : streptomycin and 50 μm 2-mercaptoethanol either with or without 5 ng/ml tumour necrosis factor-*α* (TNF-*α*; PeproTech, Rocky Hill, NJ). Cells were plated at a density of 5 × 10^4^ viable cells/well in a six-well tissue culture-treated plate (Corning Life Sciences, Amsterdam, the Netherlands). The macrophage-like RAW 264.7 cell line^29^ was cultured in RPMI-1640 supplemented with 5% fetal calf serum and 1× penicillin : streptomycin. Cell viability was determined using the CyQUANT Direct Cell Proliferation Assay Kit (Life Technologies) according to the manufacturer's instructions. Briefly, this assay is based on the assessment of both DNA content and membrane integrity. The kit comprises a fluorescent live cell-permeable nucleic acid dye in combination with a background suppression dye. The masking dye blocks the staining of dead cells and those with compromised membranes enabling only healthy cells to be stained allowing assessment of cell growth, cell viability or compound toxicity.

#### Inhibition of specific microRNAs in FL-YB cells and administration of specific microRNA mimmics

FL-YB cells were transfected using lipofectamine 2000 (Life Technologies) according to the manufacturer's protocol. Briefly, FL-YB cells were plated in six-well or 96-well tissue culture plates at a density of 4 × 10^5^ cells/ml, stimulated with 5 ng/ml TNF-*α* and incubated overnight at 37°. The medium was then removed and replaced with 1 ml/well or 50 μl/well (for six-well or 96-well plates, respectively) of complete media without antibiotics or TNF-*α*. An appropriate volume of transfection mixture containing the relevant nucleic acid was added to each well of cells and incubated at 37° for 4 hr. The transfection medium was then diluted with 2× volumes of complete media and incubated for a further 2 hr before being replaced with complete media plus 5 ng/ml TNF-*α*. Levels of specific microRNAs were depleted by transfection of cells with 100 nm of LNA-modified DNA oligonucleotide (Exiqon) with the complementary sequence to the microRNA being targeted (Table S2). Levels of specific microRNA were elevated in cells by transfection of cells with 30 nm of mirVana microRNA mimics (Ambion) (see Supporting information; Table S3). Cells were harvested 24 hr later and effects on microRNA and mRNA expression were compared.

#### Statistical analyses

Data are presented as mean ± SD. Unless otherwise indicated, differences between groups were statistically compared using Student's *t*-test.

## Results

### Effect of LTβR-blockade on FDC status in the spleen

**Figure 1:**
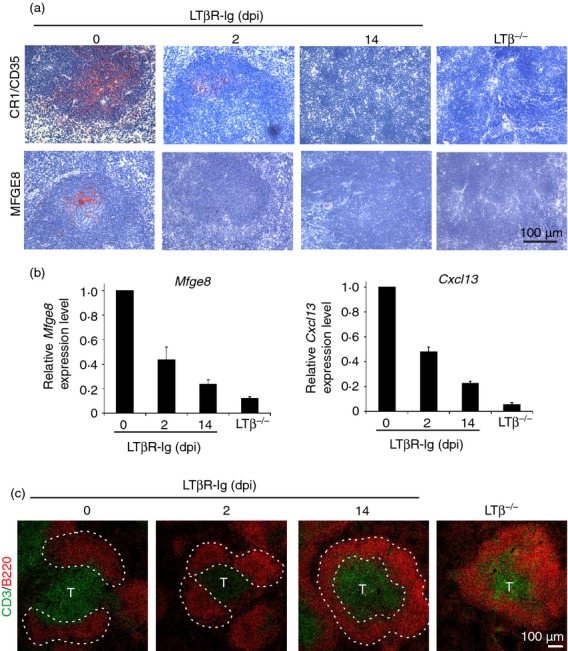
Effect of lymphotoxin *β* receptor (LT*β*R) -blockade on follicular dendritic cells (FDC) in the spleen. (a) Immunohistochemical analysis of CR1/CD35 (upper panels, red) and MFGE8 (lower panels, red) by FDC in the spleens of LT*β*^−/−^ mice and wild-type (WT) mice given a single intraperitoneal injection with LT*β*R-Ig. Sections were counterstained with haematoxylin (blue). (b) Quantitative real-time reverse transcription-PCR analysis of the effect of LT*β*R-blockade on *Mfge8*, *Cxcl13* and *Ccl19* expression in the spleen. (c) Immunofluorescent analysis of the distribution of T cells (CD3^+^ cells, green) and B cells (B220^+^ cells, red) in the spleens of LT*β*^−/−^ mice or WT mice given a single intraperitoneal injection with LT*β*R-Ig. Dpi, day post-injection with LT*β*R. Dotted lines indicate the boundaries of the B-cell areas. T, location of T-cell zone. Data are derived from spleens from four mice from each group.

Constitutive LT*β*R-mediated stimulation is required to maintain the microarchitecture of secondary lymphoid organs such as the spleen and lymph nodes.^12^,^13^,^30^,^31^ For example temporary blockade of LT*β*R-signalling by treatment of mice with soluble LT*β*R-Ig^22^ rapidly de-differentiates FDC,^12^,^13^ and this approach was used here to attempt to identify microRNAs that were potentially expressed in LT*β*R-signalling-dependent cell populations in the spleen such as FDC (Fig. 1). Immunohistochemical analysis of the spleens of LT*β*R-treated mice confirmed that within 2 days of treatment of WT mice with LT*β*R-Ig the expression of CR1 (CD35) and MFGE8 by FDC was dramatically reduced (Fig. 1). CR1- and MFGE8-expressing FDC were undetectable in the spleens of LT*β*^−/−^ mice, and as anticipated, in the spleens of WT mice by 14 days after treatment with LT*β*R-Ig (Fig. 1a). Real-time quantitative PCR analysis confirmed that the expression of *Mfge8* mRNA was also significantly reduced in the spleens of FDC-deficient LT*β*R-Ig-treated WT mice and LT*β*^−/−^ mice (Fig. 1b).

Expression of the chemokine CXCL13 by FDC is important for inducing the migration of CXCR5-expressing B cells into the FDC-containing B-cell follicles.^10^ In the absence of FDC, the expression of *Cxcl13* mRNA was significantly reduced in the spleens of LT*β*R-treated mice (Fig. 1b) and coincided with an increasing failure to form organized B-cell follicles at the later times following treatment. By 14 days after LT*β*R-treatment the B cells were situated in a continuous ring surrounding the T-cell zones^11^ (Fig. 1c). Fibroblastic reticular cells, in contrast to FDC, are stromal cells situated in the T-cell zones of secondary lymphoid organs and express high levels of cytokines and chemokines such as CCL19 which attract T cells and mononuclear phagocytes.^32^ The expression of *Ccl19* was not significantly affected by LT*β*R-blockade (Fig. 1b).

### Effect of LTβR-blockade on microRNA expression in the spleen

Next, RNA was isolated from the spleens of LT*β*^−/−^ mice and WT mice 0, 2 and 14 days after treatment with LT*β*R-Ig (four mice per group) and microRNA expression levels were compared between groups using both Affymetrix and Exiqon microRNA microarrays. Two different microarray platforms were chosen because considerable variation can exist between different platforms.^33^,^34^ Data were normalized and the microRNAs that displayed the largest reduction in expression in the LT*β*^−/−^ spleens when compared with WT controls on the EXIQON platform were identified (Table 1). Six of these 20 microRNAs were also among the most reduced microRNAs identified on the Affymetrix platform (Table 2). Furthermore, each of these microRNAs showed a dramatic reduction in their expression levels in the spleen following LT*β*R-blockade (Tables 1 and 2).

**Table 1:** Top 20 down-regulated microRNAs in the spleen after lymphotoxin *β* receptor (LT*β*R) -blockade identified by analysis on Exiqon microRNA microarrays

	LT*β*R-Ig (dpi)^1^			
MicroRNA	0	2	14	LT*β*^−/−^
**mmu-miR-100**	1·00	0·82	0·68	0·27
mmu-miR-592*	1·00	0·66	0·56	0·29
**mmu-miR-2137**	1·00	0·91	0·62	0·34
mmu-miR-3102*	1·00	0·82	0·70	0·34
**mmu-miR-762**	1·00	0·72	0·65	0·35
mmu-miR-33	1·00	1·57	0·86	0·36
**mmu-miR-138**	1·00	0·97	0·59	0·38
mmu-miR-3090*	1·00	0·86	0·75	0·40
mmu-miR-487b	1·00	1·10	1·12	0·41
mmu-miR-2861	1·00	0·81	0·63	0·42
**mmu-miR-1894-3p**	1·00	0·73	0·68	0·43
mmu-miR-25*	1·00	0·69	0·65	0·44
mmu-miR-32	1·00	1·18	0·93	0·46
**mmu-miR-187**	1·00	0·83	0·54	0·47
mmu-miR-99a	1·00	0·83	0·76	0·47
mmu-miR-10b	1·00	0·78	0·77	0·47
mmu-miR-101b	1·00	1·00	0·93	0·48
mmu-miR-711	1·00	0·94	0·76	0·48
mmu-miR-28	1·00	0·67	0·76	0·50
mmu-miR-151-5p	1·00	0·78	0·76	0·51

MicroRNAs in bold type indicate those also identified using Affymetrix microRNA microarrays.

1 Fold-change in expression level when compared with untreated wild-type mice (d 0). Dpi, day post intraperitoneal injection with LT*β*R-Ig.

**Table 2:** Top 20 down-regulated microRNAs in the spleen after lymphotoxin *β* receptor (LT*β*R) -blockade identified by analysis on Affymetrix microRNA microarrays

	LT*β*R-Ig (dpi)^1^			
MicroRNA	0	2	14	LT*β*^−/−^
mmu-miR-708	1·00	0·78	0·64	0·20
**mmu-miR-138**	1·00	0·98	0·48	0·36
**mmu-miR-2137**	1·00	0·85	0·57	0·41
**mmu-miR-187**	1·00	0·93	0·52	0·46
mmu-miR-466h	1·00	1·02	1·43	0·49
mmu-miR-1893	1·00	0·80	0·66	0·50
**mmu-miR-762**	1·00	0·76	0·59	0·50
**mmu-miR-100**	1·00	0·78	0·72	0·53
mmu-miR-1224	1·00	0·62	0·61	0·54
mmu-miR-1937a	1·00	0·92	0·79	0·56
mmu-miR-139-3p	1·00	1·20	0·77	0·56
**mmu-miR-1894-3p**	1·00	0·66	0·69	0·57
mmu-miR-1937b	1·00	0·84	0·82	0·58
mmu-miR-2133	1·00	0·99	0·63	0·60
mmu-miR-151-3p	1·00	0·84	0·73	0·61
mmu-miR-193b	1·00	1·10	0·66	0·62
mmu-miR-1959	1·00	0·59	0·68	0·63
mmu-miR-466f	1·00	1·20	1·12	0·66
mmu-miR-455	1·00	0·97	0·94	0·67
mmu-miR-19a	1·00	1·30	1·45	0·67

MicroRNAs in bold type indicate those also identified using Exiqon microRNA microarrays.

1 Fold-change in expression level when compared with untreated wild-type mice (d 0). Dpi, day post intraperitoneal injection with LT*β*R-Ig.

**Figure 2:**
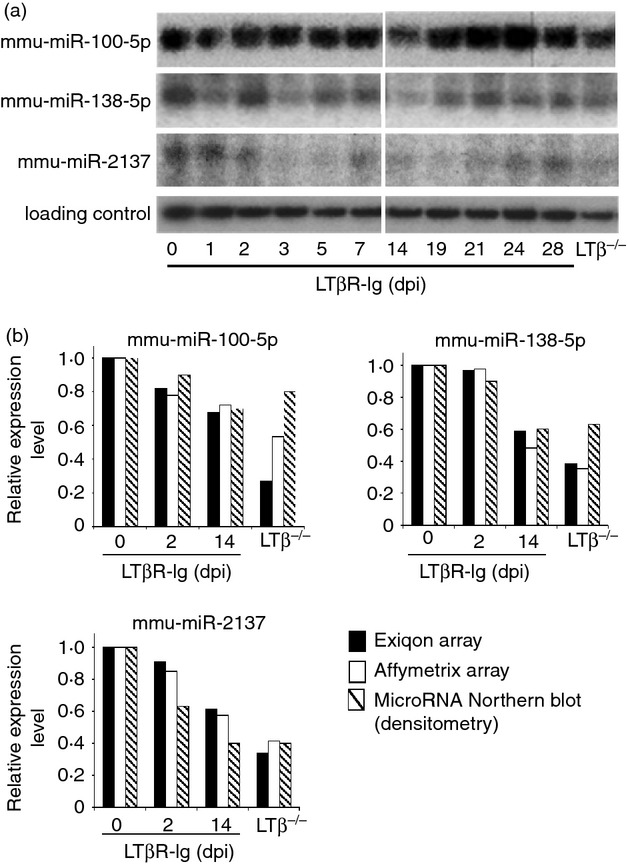
Effect of lymphotoxin *β* receptor (LT*β*R) -blockade on microRNA expression in the spleen. (a) MicroRNA Northern blot analysis of the expression of mmu-miR-100-5p, mmu-miR-138-5p and mmu-miR-2137 in the spleens of LT*β*^−/−^ mice and wild-type (WT) mice given a single intraperitoneal injection with LT*β*R-Ig. U6 snRNA was used as a loading control. (b) Comparison of the relative expression levels of each microRNA. On each histogram the relative expression levels of each microRNA derived from analysis on Exiqon microRNA microarrays (solid bars), Affymetrix microRNA microarrays (open bars), and phosphoimager quantification of the microRNA Northern blot membranes (densitometry, hatched bars) are shown. Expression levels are normalized so the mean level in untreated WT mice was 1·0. For each data point, spleens from four mice from each group were pooled into two groups of two.

The effects of LT*β*R-Ig-treatment on the expression levels of three of the microRNAs identified above (mmu-miR-100-5p, mmu-miR-138-5p and mmu-miR-2137) were then compared by microRNA Northern blot analysis. These three microRNAs were selected because they displayed the most consistent effects after LT*β*R-blockade across all samples and on each microarray platform. MicroRNA Northern blot analysis confirmed that the expression levels of each of these microRNAs was reduced in the spleens of LT*β*R-Ig-treated and LT*β*^−/−^ mice when compared with WT controls (Fig. 2).

### Expression of microRNAs by FL-YB cells

**Figure 3:**
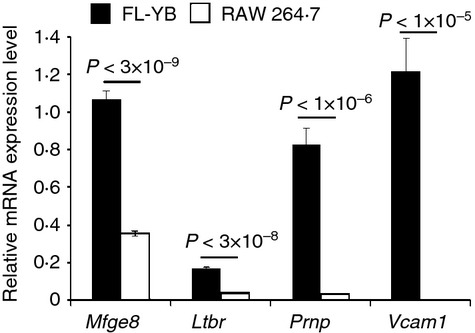
Comparison of *Ltbr*, *Mfge8*, *Prnp* and *Vcam1* expression by follicular dendritic cell (FDC) -like cell line FL-YB and the macrophage-like cell line RAW 264.7. Cells were harvested 48 hr after cultivation and RNA was extracted. Quantitative real-time reverse transcription-PCR analysis confirmed that FL-YB cells expressed *Ltbr*, *Mfge8, Prnp* and *Vcam1* highly when compared with RAW 264.7 cells. For each gene in each cell, expression levels were normalized to the expression level of *Hprt*. Data are representative of four independent experiments. Significant differences between samples were determined by Student's *t*-test.

Although effects on other LT*β*R-dependent cell populations cannot be excluded,^30^,^35^ data above suggest that mmu-miR-100-5p, mmu-miR-138-59-5p and mmu-miR-2137 are potential FDC-associated microRNAs because their expression in the spleen was reduced by 2 days after LT*β*R-blockade coincident with the rapid de-differentiation of FDC. We therefore sought to determine whether these microRNAs were expressed by FDC. The isolation of highly purified FDC from secondary lymphoid tissues is however problematic. Difficulties include: lack of truly FDC-specific cell markers, contamination with other cell types such as B cells with which they form tight clusters; contamination with macrophages, which can express high levels of MFGE8, a typical marker used to identify FDC;^1^,^8^,^36^ their low numbers within secondary lymphoid organs when compared with other cell populations; and their fastidious *in vitro* cultivation conditions that result from the FDC's requirement for constant LT*β*R stimulation to maintain the differentiated state.^12^,^13^ Therefore, to investigate the potential roles of mmu-miR-100-5p, mmu-miR-138-5p and mmu-miR-2137 in FDC, a murine FDC-like cell line, FL-YB, established from the popliteal lymph nodes of immunized BALB/c mice was used.^1^,^9^,^28^,^37^ Factors such as LT*β*R- and TNF-*α*-mediated signalling, which maintain FDC in their differentiated state *in vivo*,^12^ are also important for the proliferation of FL-YB cells.^9^,^28^ Consistent with previous data,^28^ quantitative real-time PCR analysis confirmed that FL-YB cells expressed significant levels of FDC-associated genes including *Mfge8*, *Ltbr* (which encodes LT*β*R), *Prnp* (which encodes the prion protein, PrP^C^)^38^ and *Vcam1* (which encodes vascular cell adhesion molecule 1)^39^ when compared with the macrophage RAW 264.7 cell line (Fig. 3).

**Figure 4:**
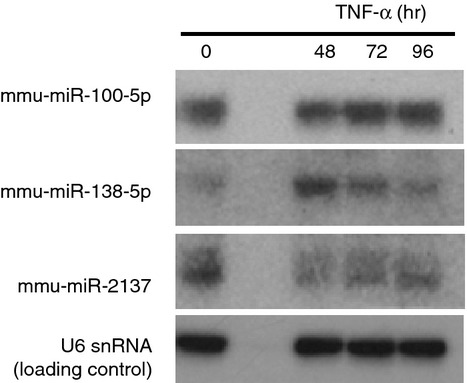
Analysis of microRNA expression in FL-YB cells. MicroRNA Northern blot analysis confirmed that FL-YB cells expressed mmu-miR-100-5p, mmu-miR-138-5p and mmu-miR-2137.

RNA was isolated from FL-YB cells at intervals following TNF-*α* stimulation and the expression of mmu-miR-100-5p, mmu-miR-138-5p and mmu-miR-2137 compared by microRNA Northern blot analysis. Expression of mmu-miR-100-5p, mmu-miR-138-5p and mmu-miR-2137 was detected in FL-YB cells (Fig. 4). Although subtle variations in the expression levels of these microRNAs were evident, each microRNA was expressed by the FL-YB cells up to at least 96 hr after cultivation.

### Effect of transient mmu-miR-100-5p inhibition on gene expression by FL-YB cells

We next used four of the many computational algorithms that have been developed to aid the identification of likely mircroRNA target genes: DIANA micro-T (http://diana.cslab.ece.ntua.gr/microT/); miRNA.org (http://www.microrna.org/microrna/home.do); miRDB (http://mirdb.org/miRDB/); RNA22 (http://cbcsrv.watson.ibm.com/rna22.html). For possibly enhancing our chances of predicting genuine targets, we required that genes were predicted as a potential target for at least two out of three microRNAs, by a minimum of three of the bioinformatics tools. However, at this level of stringency, no potential target genes were predicted for any of these three microRNAs. Consequently, using the FL-YB cells we sought to determine the effects of transient microRNA inhibition on the expression of certain key genes expressed by FDC which have been shown to influence the GC response: *Il6* (which encodes IL-6),^40^,^41^ *Ptgs1/2* (which encodes cyclooxygenase 2)^9^ and *Tlr4* (which encodes Toll-like receptor 4; TLR4).^3^,^42^

**Figure 5:**
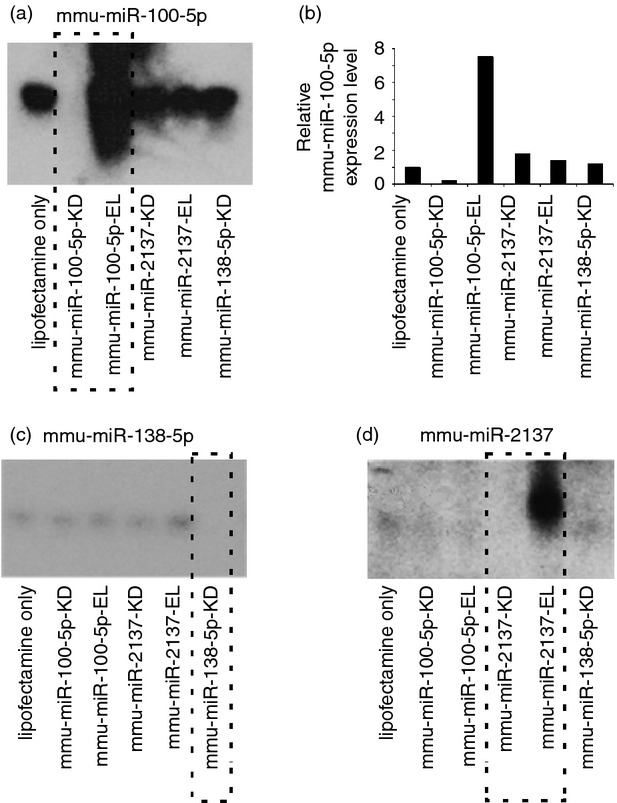
Confirmation of specific manipulation of microRNA expression levels in FL-YB cells. To deplete (knock-down, KD) microRNA levels, cells were transfected with anti-sense locked nucleic acid (LNA) oligonucleotides specific for the target microRNA. To elevate (EL) the level of specific microRNAs, cells were transfected with double-stranded RNA mimics. The effects of these manipulations on the expression levels of (a) mmu-miR-100-5p, (c) mmu-miR-138-5p and (d) mmu-miR-2137 were analysed by microRNA Northern blot analysis 24 hr later. (b) Representative phosphoimager quantification of the microRNA Northern blot membranes of the effects of these treatments on the relative expression levels of mmu-miR-100-5p in FL-YB cells. Data were normalized to the lipofectamine control. This analysis shows that the mmu-miR-100-5p-specific LNA specifically inhibited the expression of the target microRNA (mmu-miR-100-5p) by approximately fivefold and did not affect the expression levels of mmu-miR-138-5p or mmu-miR-2137. Transfection with a mmu-miR-100-59 double-stranded RNA mimic, in contrast, specifically increased the expression of this microRNA by approximately sevenfold. Data are representative of four or more independent experiments.

To deplete the available pools of specific microRNAs, FL-YB cells were transfected with anti-sense LNAs directed against mmu-miR-100-5p, mmu-miR-138-5p or mmu-miR-2137. Conversely, to increase levels of these microRNAs the cells were transfected with specific microRNA mimics. MicroRNA Northern blot analysis confirmed that each anti-sense LNA specifically inhibited the expression of the target microRNA by > 80%, whereas transfection with the microRNA mimics increased the levels of the corresponding microRNAs by at least sevenfold (Fig. 5). Transfection of the FL-YB cells with these reagents had no observable effect on cell viability (data not shown).

**Figure 6:**
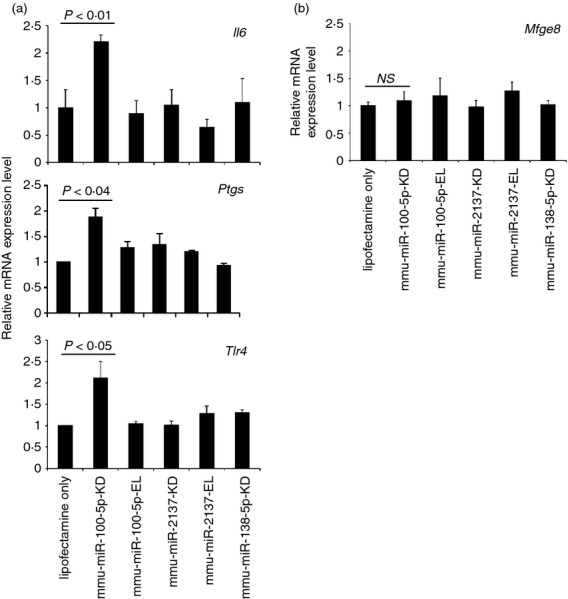
Specific inhibition of mmu-miR-100-5p enhances the expression of *Il6*, *Ptgs1/2* and *Tlr4* in FL-YB cells. To deplete (knock-down, KD) microRNA levels, cells were transfected with anti-sense locked nucleic acid (LNA) oligonucleotides specific for the target microRNA. To elevate (EL) the level of specific microRNAs, cells were transfected with double-stranded RNA mimics. RNA was isolated 24 hr later and the effects of these manipulations on the expression levels of (a) *Il6*, *Ptgs1/2*, *Tlr4* and (b) *Mfge8* mRNA were determined by quantitative real-time reverse transcription-PCR analysis. Expression levels were normalized to *Actb* and are presented relative to the levels expressed in non-transfected, lipofectamine-treated control cells. Data are representative of three or more independent experiments. Significant differences between samples were determined by Student's *t*-test. NS, not significant.

Quantitative real-time PCR analysis showed that the specific inhibition of mmu-miR-100-5p significantly enhanced the expression of three genes *Il6*, *Ptgs1/2* and *Tlr4* mRNA (Fig. 6a), while elevated levels of mmu-miR-100-5p did not affect their expression. In contrast, manipulating levels of mmu-mir-138-5p or mmu-miR-2137 did not significantly influence the expression of these three genes in FL-YB cells (Fig. 6a). Specific inhibition of mmu-miR-100-5p did not affect the expression of certain other FDC-associated genes such as *Mfge8* (Fig. 6b), *Ltbr* and *Prnp* (data not shown). Together, these data suggest that mmu-miR-100-5p may indirectly modulate the expression of *Il6*, *Ptgs1/2* and *Tlr4* mRNA in FL-YB cells.

## Discussion

LT*β*R-blockade coincided with a dramatic decrease in the expression of mmu-miR-100-5p, mmu-miR-138-5p and mmu-miR-2137 in the spleen. FDC among other cell populations in the spleen are critically dependent upon continual LT*β*R stimulation to maintain them in their differentiated state.^12^,^13^,^30^,^31^ Our data suggest that these microRNAs are potentially expressed by FDC because they were also expressed by the murine FDC-like cell line, FL-YB. Specific inhibition of mmu-miR-100-5p in FL-YB cells significantly enhanced their expression of *Il6*, *Ptgs1/2* and *Tlr4* mRNA, whereas inhibition of mmu-miR-138-5p or mmu-miR-2137 did not. The expression of each of these genes by FDC has been shown to play an important role in regulating GC size and promoting high-affinity antibody responses. MicroRNAs often act to ‘fine-tune’ the expression levels of their target genes. These data suggest that mmu-miR-100-5p may plausibly play a role in fine-tuning the expression of *Il6*, *Ptgs1/2* and *Tlr4* mRNA by FDC during GC responses.

Inhibition of miR-100-5p in FL-Y cells caused a significant increase in the expression of *Il6* mRNA. B cells within the secondary lymphoid tissues undergo GC reactions to generate high-affinity antibodies with strong antigen-specificity. In mice IL-6 plays a physiologically relevant role in generating optimal GC reactions, and promoting antibody responses, and somatic hypermutation and class-switch recombination in the immunoglobulin genes of GC B cells.^40^,^41^ As immune complex-activated FDC have been shown to produce IL-6,^43^ it is plausible that mmu-miR-100-5p may play a role in control of the GC reaction by regulating their expression of *Il6* mRNA.

Toll-like receptor 4 binds bacterial lipopolysaccharide, and signalling through this receptor promotes the expression of many pro-inflammatory genes. Oxidized phospholipids, such as those generated during apoptosis, can also act as TLR4 ligands and have been detected in FDC-containing GC.^3^ Activated FDC in mice express TLR4, and stimulation through this receptor either via lipopolysaccharide or oxidized phospholipids up-regulates their expression of adhesion molecules and Fc receptors.^3^,^42^ The absence of TLR4 expression on FDC led to a decrease in both the number and size of GC, a reduced frequency of FDC–GC B-cell interactions, reduced somatic hypermutation and a decrease in high-affinity antibody responses.^3^,^42^ Furthermore, the absence of TLR4 function on FDC alters their transcriptome impairing their expression of *Ltbr*, the adhesion molecule gene *Icam1* and cytokine genes involved in the GC reaction and antibody responses (*ll6* and *Il15*).^3^ Data presented here suggest that mmu-miR-100-5p indirectly down-regulates the expression of *Tlr4* mRNA in FL-YB cells. Hence it is plausible that the modulation of *Tlr4* mRNA by mmu-miR-100-5p in FDC may play an important role in regulating the GC reaction. TLR4 signalling up-regulates IL-6, and aberrant expression of both of these genes has been implicated in some autoimmune diseases. Up-regulation of TLR4 can induce a lupus-like autoimmune disease in mice,^44^ and expression of IL-6 is dysregulated in the serum and synovial fluid of patients with rheumatoid arthritis.^45^ Further experiments are now necessary to determine whether mmu-miR-100-5p is expressed by FDC *in vivo* and acts to fine-tune the expression of GC-related genes via modulation of *Tlr4* mRNA.

During the GC reaction, B cells undergo somatic hypermutation in the V regions of their immunoglobulin genes. Those B cells with high antigen affinity are positively selected and receive survival signals by interactions with FDC and follicular helper T cells, whereas those with low affinity do not receive these stimuli and undergo apoptosis. Data from the analysis of human^46^ and mouse^9^ FDC suggest that they express prostaglandin E_2_, which may play an immunoregulatory role in the GC. For example, its expression by FDC in association with follicular helper T-cell-derived IL-21 plays a role in the negative selection of GC B cells.^9^ Cyclooxygenase 2 (encoded by *Ptgs2*) converts arachidonic acid to prostaglandin H_2_, which can then be subsequently converted into different prostaglandins and prostacyclin.^47^ Inhibition of mmu-miR-100-5p in FL-YB cells significantly increased their expression of *Ptgs1/2* mRNA, providing a further example of how the expression of this microRNA may help to regulate the GC reaction.

Although data presented here suggest that mmu-miR-100-5p modulates the expression of *Il6*, *Ptgs1/2* and *Tlr4* mRNA, a range of target prediction algorithms failed to identify any plausible target genes. Little is known of the genes targeted by mmu-mir-100-5p, although predictions from individual algorithms include the gene encoding the mammalian target of rapamysin (*Mtor*) where this microRNA is considered to play a role in neovascularization in endothelial vascular smooth muscle cells in mice^48^ and certain tumours in humans.^49^ In the current study the expression level of *Mtor* was unchanged in the spleen after LT*β*R-blockade and was undetectable in FL-YB cells (data not shown), suggesting that the effects on gene expression described above were unlikely to be due to the targeting of *Mtor* mRNA by mmu-mir-100-5p. No likely binding sites for mmu-miR-100-5p were identified in the sequences of *Il6*, *Ptgs1/2* and *Tlr4*, suggesting that this microRNA may be indirectly influencing the expression of these genes by targeting another gene upstream of their expression.

Our data do not exclude the possibility that mmu-miR-100-5p may also modulate gene expression in other LT*β*R-dependent cell populations within secondary lymphoid tissues.^30^,^31^,^35^ Data elsewhere show that effects of LT*β*R-blockade on the presence of classical dendritic cells in the spleen were noted 10–15 days after treatment^35^ and effects on high endothelial venules in lymph nodes were evident 28 days after treatment.^30^ In the current study a decrease in the expression of mmu-miR-100-5p was evident in the spleen as soon as 2 days following LT*β*R treatment coincident with the rapid de-differentiation of FDC. Furthermore, this microRNA was confirmed to be expressed in the FDC-like cell line, FL-YB, and specific inhibition of mmu-miR-100-5p in this cell line significantly enhanced the expression of *Il6*, *Ptgs1/2* and *Tlr4* mRNA.

Currently nothing is known of the expression of microRNAs by FDC. Similarly, nothing was known of the effects of LT*β*R stimulation on microRNA expression in the spleen. These data therefore provide a useful foundation for the design of future studies aimed at determining how LT*β*R stimulation modulates microRNA expression in secondary lymphoid organs, and whether mmu-miR-100-5p helps to modulate FDC function and the GC response *in vivo*, for example by specifically manipulating microRNA expression in FDC.

## Abbreviations

CR: complement receptor
FDC: follicular dendritic cell
GC: germinal centre
IL: interleukin
LNA: locked nucleic acid
LT: lymphotoxin
LT*β*R: lymphotoxin *β* receptor
mAb: monoclonal antibody
MFGE8: milk fat globule epidermal growth factor-like factor 8
TLR4: Toll-like receptor 4
TNF: tumour necrosis factor
WT: wild-type

## Supporting Information

**Table S1.** Details of the primer sequences used for quantitative real-time PCR analysis. **Table S2.** Sequences of locked nucleic acid-modified oligonucleotides used for microRNA Northern blot analysis and specific microRNA inhibition. **Table S3.** Oligonucleotide sequences of the microRNA mimics used.
