# Supplementary material for: MicroRNA-100-5p indirectly modulates the expression of Il6, Ptgs1/2 and Tlr4 mRNA in the mouse follicular dendritic cell-like cell line, FL-Y
Source: Immunology. 2014 Dec 8;144(1):34–44. doi: 10.1111/imm.12342 (PMC4264908; doi:10.1111/imm.12342)
Supplement: Supplementary file 1 [file imm0144-0034-sd1.pdf]

Supplementary Table 1. Details of the primer sequences used for quantitative real-time PCR analysis.

Gene symbol	Primer	Primer sequence
Actb	Forward	TGACAGGATGCAGAAGGAGA
	Reverse	GTACTTGCGCTCAGGAGGAG
Hprt	Forward	GCAGTACAGCCCCAAAATGG
	Reverse	AACAAAGTCTGGCCTGTATCCAA
Cxcl13	Forward	ACAGACTCCGAGCTAAAGGTTG
	Reverse	AATGGGCTTCCAGAATACCG
Ccl19	Forward	GATCGCATCATCCGAAGACT
	Reverse	GAGGCCTGGTCCTCTCTTCT
Mfge8	Forward	TTCTGTGACTCCAGCCTGTG
	Reverse	GGAGCATGGTCCTCTCTCAG
Tlr4	Forward	ACTCTGATCATGGCACTGTTCTT
	Reverse	GCTCAGATCTATGTTGGTTGA
Vcam-1	Forward	CCGGCATATACGAGTGTGAA
	Reverse	GGAGTTCGGGCGAAAAATAG
Ltbr	Forward	CAGAGAGCTGGAGGCTGAAC
	Reverse	TATGTAGATGTTGCCGGTGA
Il-6	Forward	CAAAGCCAGAGTCCTTCAGAG
	Reverse	GCCACTCCTTCTGTGACTCC
Ptgs1/2	Forward	TGTACAAGCAGTGGCAAAGG
	Reverse	TTCTGCAGCCATTTCCTTCT
Prnp	Forward	CTGAAGCATTCTGCCTTCCT
	Reverse	GCCGACATCAGTCCACATAG

Supplementary Table 2. Sequences of LNA-modified oligonucleotides used for microRNA Northern blot analysis and specific microRNA inhibition.

**miRNA target**	**Sequence**
mmu-miR-2137	CT+CCC+TGG+GGC+TCC+CGC+CGG+C
mmu-miR-138-5p	C+GGC+CTG+ATT+CAC+AAC+ACC+AGCT
mmu-miR-100-5p	C+ACA+AGT+TCG+GAT+CTA+CGG+GTT

Supplementary Table 3. Oligonucleotide sequences of the microRNA mimics used.

**Target miRNA**	**Sequence**
mmu-miR-100-5p	AACCCGUAGAUCCGAACUUGUG
mmu-miR-2137	GCCGGCGGGAGCCCCAGGGAG
